# A Snapshot of Histone Modifications within Transposable Elements in *Drosophila* Wild Type Strains

**DOI:** 10.1371/journal.pone.0044253

**Published:** 2012-09-04

**Authors:** Rita Rebollo, Béatrice Horard, Flora Begeot, Marion Delattre, Eric Gilson, Cristina Vieira

**Affiliations:** 1 Laboratoire de Biométrie et Biologie Evolutive, UMR5558, CNRS, Université de Lyon, Université Lyon 1, Villeurbanne, France; 2 Institute of Research on Cancer and Aging, CNRS UMR7284/INSERM U1081/UNS Faculté de Médecine, Nice, France; 3 Département de Génétique et Evolution, Université de Genève, Genève, Switzerland; North Carolina State University, United States of America

## Abstract

Transposable elements (TEs) are a major source of genetic variability in genomes, creating genetic novelty and driving genome evolution. Analysis of sequenced genomes has revealed considerable diversity in TE families, copy number, and localization between different, closely related species. For instance, although the twin species *Drosophila melanogaster* and *D. simulans* share the same TE families, they display different amounts of TEs. Furthermore, previous analyses of wild type derived strains of *D. simulans* have revealed high polymorphism regarding TE copy number within this species. Several factors may influence the diversity and abundance of TEs in a genome, including molecular mechanisms such as epigenetic factors, which could be a source of variation in TE success. In this paper, we present the first analysis of the epigenetic status of four TE families (*roo, tirant, 412* and *F*) in seven wild type strains of *D. melanogaster* and *D. simulans*. Our data shows intra- and inter-specific variations in the histone marks that adorn TE copies. Our results demonstrate that the chromatin state of common TEs varies among TE families, between closely related species and also between wild type strains.

## Introduction

Transposable elements (TEs) are a major source of genetic novelty and genome evolution [1], [2]. TEs are mobile sequences that may induce mutations through mobilization and recombination, often in a specific, individual manner [3]. Furthermore, TEs may provide new genetic features [4]–[6] and regulatory sequences [4], [6]–[8] contributing to the formation and remodeling of host gene networks [9], [10]. TEs can be classified into two distinct groups, DNA transposons and retrotransposons, which will move either through a DNA molecule or an RNA intermediate respectively. We focused this study on retrotransposons with families presenting long terminal repeats (LTR retrotransposons) in their extremities or not (non-LTR retrotransposons).

The factors that govern intra- and inter-species TE diversity are complex. They consist of a combination of the intrinsic properties of the TEs themselves (e.g. transposition mechanism, infectivity), the properties of the host’s ecology (e.g. effective size and structure of the populations), and those of the genome (e.g. TE regulation, gene density, genome size). TEs are maintained in the genome, but are usually silenced via molecular mechanisms that protect the genome against the deleterious effects of transposition and/or recombination, whilst preserving the possibility of creating variability, for example, in response to an environmental stress. Epigenetic regulation is one of the molecular mechanisms that controls TE expression and/or activity through semi-redundant pathways including histone post-translational modifications, DNA methylation, and the production of non-coding small RNAs [11], [12]. Histone post translational modifications have been associated with permissive and repressive chromatin states. Transposable elements, frequently found in heterochromatic domains, are often described as being associated with repressive histone marks. In SETDB1 knock out mice an enzyme responsible for the repressive H3K9me3 modification, induces over expression of specific LTR retrotransposon families [13], [14]. Furthermore, different classes of retrotransposons are upregulated in lysine-specific demethylase mouse mutants (LSD1/KDM1A), suggesting an adaptation of the epigenetic regulatory mechanisms to the type of transposable element family. In plants, H3K9me3 and H3K27me1 are involved in TE silencing [15]. In Drosophila, the constitutive heterochromatin is enriched in H3K9me2, whereas facultative heterochromatin is preferentially labeled by H3K27me3 [16], [17]. While studying the heterochromatin-euchromatin boundary, Yasuhara et al. showed that TEs are associated with H3K9me2 in *D. melanogaster* embryos and high copy number TEs, such as the LTR retrotransposon *roo,* have lower H3K9me2 enrichment [18]. In Drosophila somatic tissues different retrotransposons are associated with H3K9me3 and H3K9me2 in both their promoter regions and open reading frames [19]. Interestingly, H3K4me2 is observed along with the previous repressive marks, in both promoter and ORF of the HET-A LTR retrotransposon [19]. Association of both repressive (H3K9me2/3) and permissive (H3K4me2/3) histone marks was also observed in retrotransposons found in both euchromatin and heterochromatin regions, although the enrichment for H3K4me2/3 is weak or moderate in the latter [20], [21]. In addition to the complex association of histone marks and TEs observed in Drosophila, there is evidence that distinct chromatin patterns might be observed not only between different TE families as noted above, but also within a given TE family [22], [23]. Therefore, the histone modifications associated with TEs in Drosophila are still poorly understood, and are rarely discussed in the literature.

Drosophila has fewer TEs than other organisms, such as humans;15% of the Drosophila genome is composed by TEs versus 50% for humans [1]; but has a high level of TE activity, as demonstrated by the large number of spontaneous mutations that are attributed to TE movements, and by the high number of full-length TEs found in the sequenced genome of *D. melanogaster* [24]–[26]. Drosophila contains putative active elements [27], and hence is an interesting model for studying the impact of TEs on genetic variability and genome evolution. *D. melanogaster* and *D. simulans* contain the same TE families, with more than 90% of sequence identity in most cases [28]. However, an over-representation of almost all TEs is observed in *D. melanogaster* [29], as shown by the sequenced genome analysis of both species. This study estimates that euchromatic TEs account for ∼5% and 2% of the genome in *D. melanogaster* and *D. simulans* respectively [30].

Investigation of TEs and associated histone modifications has never been carried out in a natural population of Drosophila. This restricts our understanding of the mechanisms that control TE behavior and dynamics in genomes to a static view. Wild type derived strains of natural populations of both *D. melanogaster* and *D. simulans* provide an excellent model system to investigate these questions. Such strains have been collected from different geographic locations in the last 30 years and have been maintained as inbred lines in the laboratory. Copy numbers of TEs are relatively homogeneous in wild type strains of *D. melanogaster*, since high numbers of copies are present in all the strains analyzed [29]. In contrast, wild type strains of *D. simulans* are highly variable; a high copy number of a given element may be observed in one strain, with no copies in another strain [29]. These observations were based on counting the TE copy number through polytene chromosome in-situ hybridization experiments in which TEs of centromeric, telomeric and dense heterochromatic regions cannot be counted individually [29]. Therefore, the variations in copy number observed between wild type strains of *D. melanogaster* and *D. simulans* reflect only euchromatic copies. Such differences suggest different levels of TE regulation or population biology in both species.

**Figure 1:**
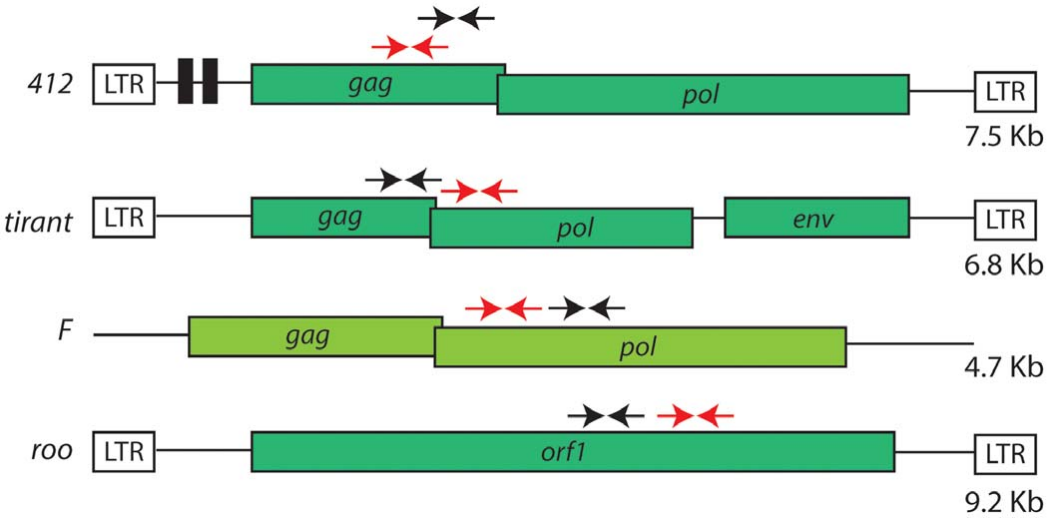
Cartoon of the four retrotransposons studied (not to scale). Colored boxes represent open reading frames (ORF) and white boxes long terminal repeats (LTR). Arrows represent the quantitative PCR primers used for ChIP-qPCR (black arrows) and expression analysis (red arrows). The size of each canonical element is given.

In order to better characterize the histone modifications associated with specific TE families, we studied all retrotransposon families that present full length copies in both *D. melanogaster* and *D. simulans* species : *roo*, *tirant*, *412* (LTR retrotransposons) and *F* (non-LTR retrotransposon), shown in Figure 1. Seven wild type strains of *D. melanogaster* and *D. simulans* were assayed for the typical histone post translational modifications described above (H3K9me2, H3K27me3 and H3K4me2) and RNA steady state level. We observed variable histone patterns between both species and wild type strains, and between different TE families. We also observed RNA transcript variation among strains and species. The complex pattern that we observed with no fixed associations between histone marks and TEs suggest that the activity of TEs may be uncoupled with the histone marks, and that a few specific copies of TEs may be responsible for most of the observed TE activity.

## Results

### Transposable Elements are Associated with Different Histone Marks

**Figure 2:**
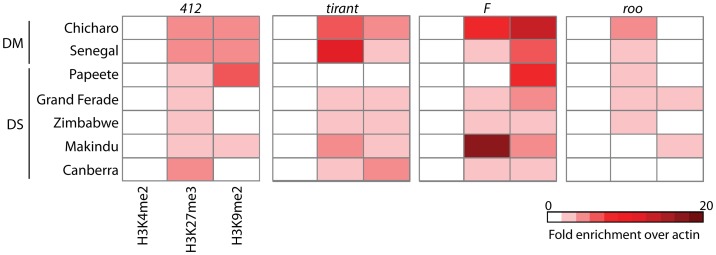
Histone post-translational modifications associated with Drosophila transposable elements. Heatmap of ChIP-qPCR average fold enrichment for all TEs analyzed. Fold enrichment for each wild type strain is normalized by input, hence copy number, and also by *actin,* allowing comparison of species and srains. Each row represents a wild type strain of either *D. melanogaster* (DM) or *D. simulans* (DS). Each column represents a different antibody used (H3K4me2, H3K27me3 and H3K9me2). Location of primers used for ChIP-qPCR amplification is shown in Figure 1.

In order to study the chromatin environment of different transposable elements in several wild type strains of both *D. melanogaster* and *D. simulans,* we performed cross-linked chromatin immunoprecipitation (X-CHIP) with antibodies specific for euchromatin (H3K4me2), facultative heterochromatin (H3K27me3) and constitutive heterochromatin (H3K9me2) in two to four biological replicates of late embryos for seven wild type strains of Drosophila. Quantitative PCR fold enrichment for all histone post-translational modifications was calculated relative to input and therefore normalized by copy number, and also by actin in order to compare species and wild type strain data (Figure S1). We have also tested our protocol with known targets of the three histone marks (Figure S2). Histograms of fold enrichment for each ChIP experiment for each wild type strain and TE studied are shown in Figure S3. Our analysis showed a lack of H3K4me2 associated with TEs and variable levels of both heterochromatic marks studied (Figure 2). As we and others have already described association of TE copies with H3K4me2 [20], [22], we hypothesize that the frequency of such association is very rare and therefore can't be observed in a bulk analysis. Indeed, Riddle et al. (2011) have shown through ChIP-seq in S2 cells (embryonic derived cells) that while four copies of *tirant* show an average enrichment for H3K4me2, 35 copies lack such mark [20]. It is worth mentioning that H3K4me2 is described in both the promoter (LTRs) and internal regions of some TEs [20] implying no bias generated by our choice of primers within internal regions of the copies studied (Figure 1). Furthermore, not all TEs are associated with heterochromatic histone modifications. *Roo* copies are mainly devoid of H3K9me2, suggesting lack of this element from dense heterochromatic regions (Figure 2, Mann Whitney test within each species showed significant depletion of H3K9me2 for *roo* with a p-value <0.05). Indeed, *roo* is the most abundant element in both *D. melanogaster* and *D. simulans* chromosomal arms [29], which represents Drosophila euchromatin. Our analysis confirmed our previous observation that *tirant* copies can be highly associated with H3K27me3 [22] (Figure 2). *F* copies are consistently associated with heterochromatic marks in *D. melanogaster* and in *D. simulans* (Figure 2). *412* elements harbor no distinct pattern of histone modifications and some strains lack all histones modifications assayed. Our analysis is in agreement with previous observations, suggesting that TEs in Drosophila have a complex chromatin signature, with the presence of different heterochromatic and euchromatic marks [20].

### *D. melanogaster* and *D. simulans* Show Different Levels of H3K9me2 and H3K27me3 in TEs

**Figure 3:**
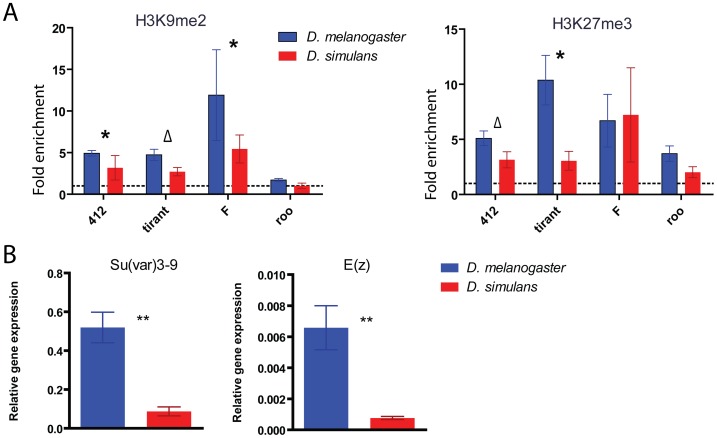
*D. melanogaster* and *D. simulans* display differences in TE chromatin state and expression of epigenetic writers. A. Fold enrichment of H3K9me2 (left panel) and H3K27me3 (right panel) for the four transposable elements studied are depicted per species (mean ± SE). *Roo* copies show a significant lack of H3K9me2 (Mann Whitney test, p-value <0.05). Histone fold enrichment of each TE family was compared between species and the results of the Mann Whitney test are shown (p-value * <0.05 and Δ <0.1). B. Quantification of RNA steady-state levels of the methyltransferases responsible for H3K9me2 and H3K27me3 deposition (mean ± SE). *D. melanogaster* (blue), *D. simulans* (red). Mann Whitney P-values between species are shown with asterisks (p-value ** <0.001).

*D. melanogaster* possess more TEs than *D. simulans* [29] suggesting that the relationship between TEs and the host genome is different between these species. By comparing fold enrichment of H3K9me2 and H3K27me3 of *412, tirant* and *F* between both species we observed a trend of higher association of these heterochromatic marks with *D. melanogaster* TE families than with *D. simulans* (Figure 3A). TEs belonging to the same TE family are therefore present more frequently within heterochromatic regions in *D. melanogaster* than in *D. simulans*. We also compared the steady-state level of methyltransferases responsible for epigenetic modifications or interactions in both species (Figure S4). Only the H3K9me2 and H3K27me3 methyltransferases, Su(var)3–9 and E(z) respectively, show significantly higher expression in *D. melanogaster* compared to *D. simulans* (Figure 3B). Differences in expression of epigenetic modifiers have previously been related to differences in downstream targets, as shown by a decrease in expression of genes responsible for small RNA production and heterochromatin formation in *Arabidopsis thaliana* pollen which correlates with demethylation and activation of TEs [31]. Therefore, decrease in expression of the “writers” of H3K9me2 and H3K27me3 could be responsible for a decrease in the frequency of these marks in *D. simulans*.

### Expression Levels of TEs in *D. melanogaster* and *D. simulans*

**Figure 4:**
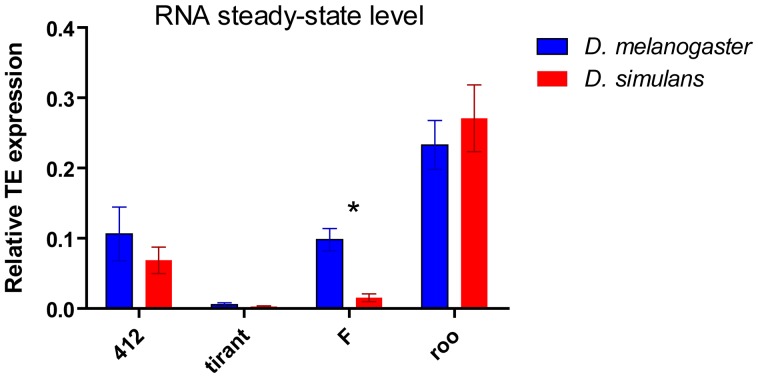
Expression of transposable element families in *D. melanogaster* (blue) and *D. simulans* (red). Mann Whitney P-values are shown with asterisks (p-value *<0.05).

We wanted to investigate whether the histone marks associated with bulk TEs in both species correlates with overall expression of the same TE families. We quantified the RNA steady state levels of all the TE families in the seven wild type strains. Overall, the level of expression of the four TE families was very different. Not surprisingly, *roo* was highly expressed in both species, correlating with the lack of heterochromatic histone marks within *roo* copies (Figure 4, Mann Whitney test p-value of *roo* expression compared to the other TE families is <0.05 for *D. melanogaster* and <0.001 for *D. simulans*). *Tirant* presented a lower steady-state level of RNA compared to the other TEs (Mann Whitney p-value <0.05 for *D. melanogaster* and <0.001 for *D. simulans*). Interestingly, expression levels of most of the TEs are equivalent between both species, with the exception of the *F* element which shows a higher RNA steady-state level in *D. melanogaster* populations compared to *D. simulans*, while both species present dense heterochromatic copies. Hence, there is no correlation between decrease in global H3K9me3 along with H3K27me3 and lower methyltransferase expression with TE family expression in *D. simulans*. Our current analyses did not allow us to assay for single copy histone modifications or transcript levels. Hence, it is important to note that a single copy may be responsible for all the transcripts quantified and harbor a chromatin state different from the global trend observed in Figure 2. In order to estimate the percentage of active copies, assuming all copies could produce transcripts, we have quantified the number of copies for each TE family with the same primers used for RNA quantification (Figure S5). Only *F* and *roo* presented significantly higher RNA steady-state level per copy in *D. melanogaster* and *D. simulans* respectively, again, uncoupled with the histone marks present within both TE families.

### Variation of TE Histone Marks and Expression between Wild Type Strains

We next compared the RNA steady-state level of each TE family along with enrichment for histone marks across wild type strains from populations of *D. simulans*. The *D. melanogaster* dataset only harbored two wild type strains which, in our view, is not representative of the variability that may exist within a species. We found a significant variation in TE expression between wild type strains of *D. simulans* (Kruskal Wallis p-value <0.05) however, no significant variation in histone post-translational modifications was observed. Variation in wild type strains of TE expression, copy number, and histone modifications showed no significant correlation, with the exception of *roo* (Pearson p-value <0.05 between copy number and expression). Hence, the variation in expression levels observed in our analysis within *D. simulans* cannot be explained by TE chromatin state or copy number.

## Discussion

Several studies have suggested that the epigenetic regulatory system might be flexible [32]. The epigenetic polymorphism observed between twin brothers [33] and the presence of different gene methylation patterns in different *Arabidopsis thaliana* ecotypes [34] exemplify natural epigenetic variation. In addition, large differences in TE copy numbers are often observed between closely-related species [35], [36]. These observations suggest that, although in most circumstances TEs are efficiently silenced, the balance between TE activity and silencing can be altered in the wild. In order to understand TE dynamics, it therefore appears essential to integrate epigenetic and natural population analyses, as many authors have recently pointed out [32], [37], [38]. Nevertheless, the study of variation in natural populations harbors intrinsic difficulties due to variation in genetic background, environment, global and single effects on expression etc. Here, we have attempted to shed light on the epigenetic variation that may exist within transposable elements of different wild type strains. We have demonstrated that TE families possess different chromatin states, such as the *roo* element, which is the only TE in our analysis constantly devoid of constitutive repressive marks (H3K9me2). *Roo* is highly expressed and abundant in both *D. melanogaster* and *D. simulans* species. Hence, *roo* is the only TE family to present a clear histone and expression profile, and is the only profile common to both species. We have confirmed the recent findings that the pattern of histone modifications associated with Drosophila TEs is more complex than in other species [20]. We have also shown that two close species, *D. melanogaster* and *D. simulans,* harbor distinct patterns of TE expression and chromatin state. For all TEs analyzed, *D. melanogaster* has an average higher copy number and expression but TEs are also highly associated with repressive histone marks. Interestingly, the expression of writers of such heterochromatic marks, Su(var)3–9 and E(z), is nearly absent in *D. simulans* wild type strains, where TEs are less abundant than in *D. melanogaster*. The biological meaning of this disparity remains unknown. Finally, we found no significant correlation between the expression, chromatin state or copy number of TEs in wild type strains of *D. simulans*. We have previously shown that different copies of the same TE family can harbor distinct histone post-translational modifications [22]. Therefore, it is extremely plausible that the data discussed here is a reflection of the majority of the copies found in the genome, but in no way a detailed description of the epigenetic marks that TE copies may harbor in Drosophila. The master copy hypothesis postulates that only one active copy is necessary to maintain retrotransposition [39]. Nevertheless, very often, more than one full length copy is observed in the genomes and truncated copies that do not retrotranspose are still able to produce transcripts. Therefore, the complete transcriptome of a transposable element is complex and hard to understand. By studying single copy epigenetic modifications one can infer which copies are in a permissive state and capable of producing transcripts and which copies are not. Riddle et al 2011 [20] show that each TE family has a very complex epigenetic environment and very often, only a small percentage of the entire population of TEs harbor polymerase binding and permissive marks as H3K4me3. While such analysis may be more complex in *D. melanogaster* strains since the number of TE copies is high, *D. simulans* wild-type strains provide an excellent work model since they have lower copy number and fewer full-length putative active copies. Indeed, we have recently annotated these TE families and others in the *D. simulans* sequenced genome, and most of the copies are internally deleted in this species [24]. However, the four TE families analyzed in this study present full-length elements and therefore putatively active copies in the *D. simulans* sequenced genome. We are currently trying to map all the copies from the four TEs analyzed here in the seven wild type strains, enabling comparison between common copies and insertionally polymorphic copies in different strains. While such analysis will give us a better view of the chromatin marks present in one strain, it will not elucidate the lack of correlation between TE expression and chromatin state as TEs are highly similar in Drosophila and hence transcripts are difficult to map at one single copy.

Since laboratory breeding conditions are equal for all the strains, one could suggest that the original epigenetic differences between strains may no longer exist. However, we do observe such differences, suggesting that the laboratory conditions do not lead to an equivalent epigenome. We cannot assume that such differences arise with the inbreeding of the wild-type derived strains in the laboratory or are original epigenetic differences, maintained during breeding. Experiments using fresh collections of Drosophila populations should answer such question. While this report demonstrates the importance of studying natural populations, the perfect model system where one can control all the parameters is still not available, especially for modeling *D. simulans* populations.

## Materials and Methods

### Wild Type Strains

Work has carried out on wild type derived strains originally collected from several geographical regions [40]. Wild type strains of *D. melanogaster* were collected from Portugal (Chicharo, 1994) and Senegal (1994). The wild type strains of *D. simulans* were from Kenya (Makindu, 1988), Zimbabwe (1991), Australia (Canberra, collection date not known), French Polynesia (Papeete, collection date not known) and France (Grand Ferrade, 1992). These populations were maintained in the laboratory as isofemale lines or small mass cultures with around 50 pairs in each generation. Stock flies were maintained at 24°C.

### Estimation of the TE Copy Number

DNA was extracted in triplicate from 14 h to 16 h embryos using the DNAeasy blood and tissue kit (Qiagen), for each wild type strain. Genomic DNA was diluted to 0.16 ng/µl. Linear real time PCR was performed using Power SYBR Green Master Mix (Applied Biosystems), on a SDS 7900 HT instrument (Applied Biosystems) with the following parameters: 50°C for two minutes, 95°C for ten minutes, 45 cycles of 95°C for 15 seconds and then 60°C for one minute. Each genomic region was tested with specific primers designed using the Primer Express v 2.0 program (Applied Biosystems) using default parameters. Primer sequences are given in Table S1. All primer efficiencies were calculated using standard dilutions of both *D. melanogaster* and *D. simulans* samples, and were comprised between 1.9 and 2.0. All primer efficiencies were equivalent. Triplicate samples were taken, PCRs were performed in triplicate, and results obtained for each primer set tested were normalized using three control genes treated in parallel (*rp49, RNApol II, EFG1*). Raw Ct values were obtained with SDS 2.2 (Applied Biosystems) and normalization factor and fold changes were calculated using the GeNorm method [41]. Real-time PCR and data analysis were performed using the Genomics Platform, NCCR “Frontiers in Genetics” (http://www.frontiers-in-genetics.org/genomics.htm). All statistical analyses in this report were carried out using GraphPad prism.

### Quantification of the TE Expression

RNA was isolated from 14 h to 16 h embryos. RNA was extracted using TRIzol® reagent (Invitrogen), according to the manufacturer’s instructions, followed by chloroform/isoamyl-alcohol (24∶1) purification. Following DNase treatment (Ambion DNA free™), the OD260/280 (interval 1.9–2.0) values of the RNA samples were determined by spectrophotometry. The integrity of the RNA was assessed by Agilent 2100 Bioanalyser (Agilent Technologies Inc, Palo Alto CA). cDNA was prepared from 1 µg total RNA, using random hexamers and the Supercript II reverse transcriptase (Invitrogen). Further analysis of transcripts was carried out using quantitative PCR as described above (including primers). Figure S1 shows equivalent levels of control genes between the species and wild type strains.

### Transcription of Epigenetic Factors

Total RNA was extracted from 14 h to 16 h embryos from all the wild type strains analyzed (two biological replicates for each strain) with the RNeasy Protect Mini Kit (Qiagen). 1 µg of total RNA was treated with Ambion's DNAse kit. ThermoScript RT-PCR system (Invitrogen) was used to synthesize two different cDNAs (55°C for 90 min and 85°C for 5 min). A retrotranscriptase-free reaction was used as a negative control against DNA contamination. Total cDNA was synthesized with a mix of oligo-dt/random primers (1∶1). cDNA samples were diluted 80 fold, and PCR was carried out using Power SYBR Green Master Mix (Roche) on the LightCycler (Roche) using specific primers for each enzyme analyzed. Primers were chosen surrounding introns in order to amplify 150–250 bp fragments of cDNA (see Table S1). Genes analyzed were chosen as being part of the chromatin remodeling processes, such as *Su(var)3–9, Ash1, E(z), HP1, HDAC3,* and *Su(var)4–20*. All primers displayed equivalent efficiency (between 1.9 and 2.0) calculated through standard dilutions of *D. melanogaster* and *D. simulans* samples. Quantitative PCR cycling conditions were 5 min at 95°C (1 cycle), 15 s at 95°C, 10 s at 60°C, 20 s at 72°C (50 cycles). Reactions were performed in duplicate, and standard curves calculated from serial dilutions of specific amplified PCR fragments. Quantity of transcripts was estimated relative to *rp49* and *18* *sRNA* expression. Relative quantification was calculated as described above for TE expression.

### Histone Modifications within TEs

Extraction of chromatin from 14 h to 16 h embryos and immunoprecipitation were adapted from Sandmann et al. 2006 [42]. Cell lysis buffer was changed to 5 mM PIPES pH 8, 85 mM KCl, 0.5% Nonidet P-40 supplemented with protease inhibitors. Chromatin was disrupted with a Bioruptor sonicator water bath (Diagenode, Liège, Belgium) for 6 X (30-s on/30-s off) cycles at high power to generate random fragments from 1 kb to 500 bp. Chromatin was incubated overnight at 4°C with antibodies recognizing H3K9me2 (Millipore, Billerica, MA, USA; 07441), H3K27me3 (Millipore 07449), H3K4me2 (Millipore 07030), H3 (Abcam, Cambridge, UK; ab1791), or rabbit IgG (Sigma-Aldrich, St. Louis, MO, USA; I5006). The antigen-antibody complexes were washed as previsouly described [42], with the second washing solution as follows: TE 2X, 500 mM NaCl, 1% Triton, 0.1% SDS. To quantify each IP, real-time PCR was performed using QuantiTect SYBR Green PCR kit (Qiagen) on a MXP3000P PCR system (Stratagene, La Jolla, CA, USA). Reactions were performed in duplicate, and standard curves calculated using serial dilutions of input chromatin. To evaluate the relative enrichment of each TE after IP, we calculated the difference in cycles between the IP-enriched sample and the input DNA for TE copies and for a control (*actin*-CG4027). Primer sequences were selected from the coding region for each TE (Figure 1 and Table S1). All primers displayed equivalent efficiency (between 1.9 and 2.0) calculated using standard dilutions of *D. melanogaster* and *D. simulans* samples. The heatmap was drawn using a free online software available here http://www.chibi.ubc.ca/matrix2png/bin/matrix2png.cgi. Average enrichment for all histone marks was calculated based on all qPCR results (Figure S3).

## Supporting Information

Figure S1 **Ct (cycle threshold) comparison between** ***D. melanogaster*** **(blue) and** ***D. simulans*** **(pink) reference genes and TEs.** For all experiments (RT-qPCR, qPCR (A) and ChIP(B)) reference genes used for both species are either equally transcribed, or equally associated with post translational histone modifications. Such data allowed us to compare wild type strains of *D. simulans* with those of *D. melanogaster* as previously described [43].
(PDF)

Figure S2 **Positive controls for ChIP antibodies.** A. H3K4me2 is enriched in the *rp49* promoter. B. Satellite *1.688* is enriched in H3K9me2 as expected. Low H3K27me3 is observed as expected with dense heterochromatic regions. All chromatin immunoprecipitations were carried out using *D. melanogaster* Chicharo strain.
(PDF)

Figure S3 **ChIP fold enrichment and transcript analysis for each wild type strain and TE studied (mean ± SE).** RT-qPCR data for *D. melanogaster* (blue) and *D. simulans* (red). ChIP analysis (black), a dotted line is shown at 1 for no enrichment relative to *actin*. DM : *D. melanogaster*, DS : *D. simulans*.
(PDF)

Figure S4 **Quantification of RNA steady-state level of epigenetic related enzymes (mean ± SE).** *D. melanogaster* (blue), *D. simulans* (red). Mann Whitney p-values are shown with asterisks (p-value ** <0.001).
(PDF)

Figure S5 **Quantification of TE RNA steady-state level, normalized by copy number (mean ± SE).** *D. melanogaster* (blue), *D. simulans* (red). Mann Whitney p-values are shown with asterisks (p-value *<0.05, ** <0.001).
(PDF)

Table S1 **Primer sequences.**
(XLSX)
