# Supplementary figures and images for: A Snapshot of Histone Modifications within Transposable Elements in Drosophila Wild Type Strains
Source: PLoS One. 2012 Sep 4;7(9):e44253. doi: 10.1371/journal.pone.0044253 (PMC3433462; doi:10.1371/journal.pone.0044253)

**A****Expression analysis**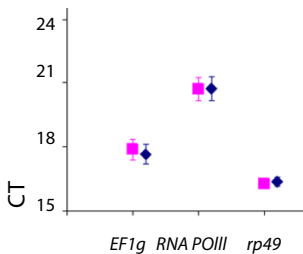**B****ChIP**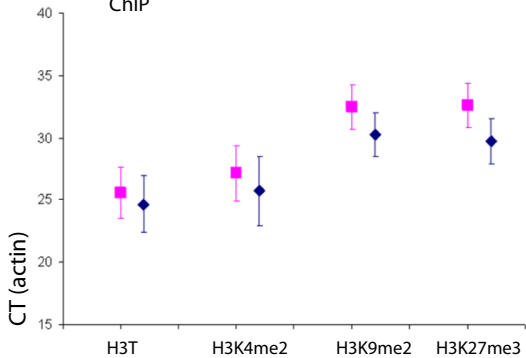

Supplement: Figure S1 — Ct (cycle threshold) comparison between D. melanogaster (blue) and D. simulans (pink) reference genes and TEs. For all experiments (RT-qPCR, qPCR (A) and ChIP(B)) reference genes used for both species are either equally transcribed, or equally associated with post translational histone modifications. Such data allowed us to compare wild type strains of D. simulans with those of D. melanogaster as previously described [43]. (PDF) [file pone.0044253.s001.pdf]

A

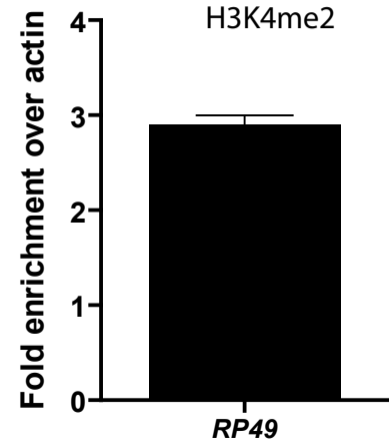

B

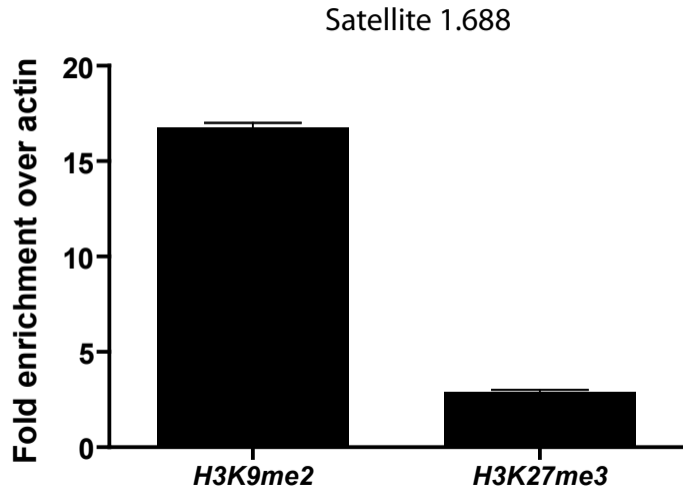

Supplement: Figure S2 — Positive controls for ChIP antibodies. A. H3K4me2 is enriched in the rp49 promoter. B. Satellite 1.688 is enriched in H3K9me2 as expected. Low H3K27me3 is observed as expected with dense heterochromatic regions. All chromatin immunoprecipitations were carried out using D. melanogaster Chicharo strain. (PDF) [file pone.0044253.s002.pdf]

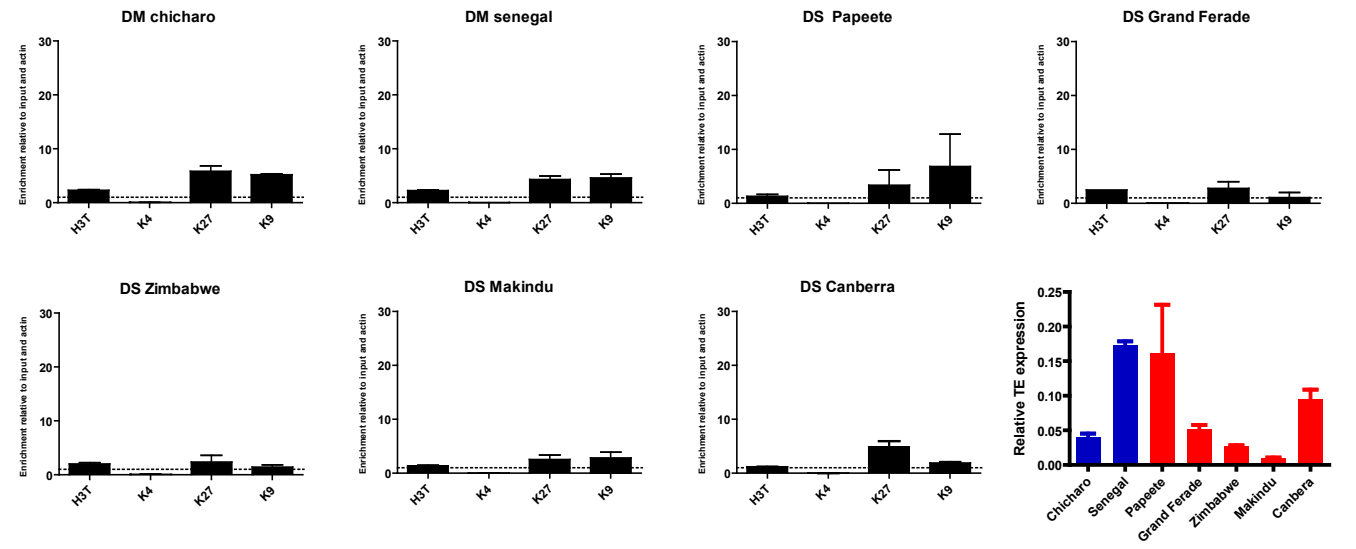

*tirant*

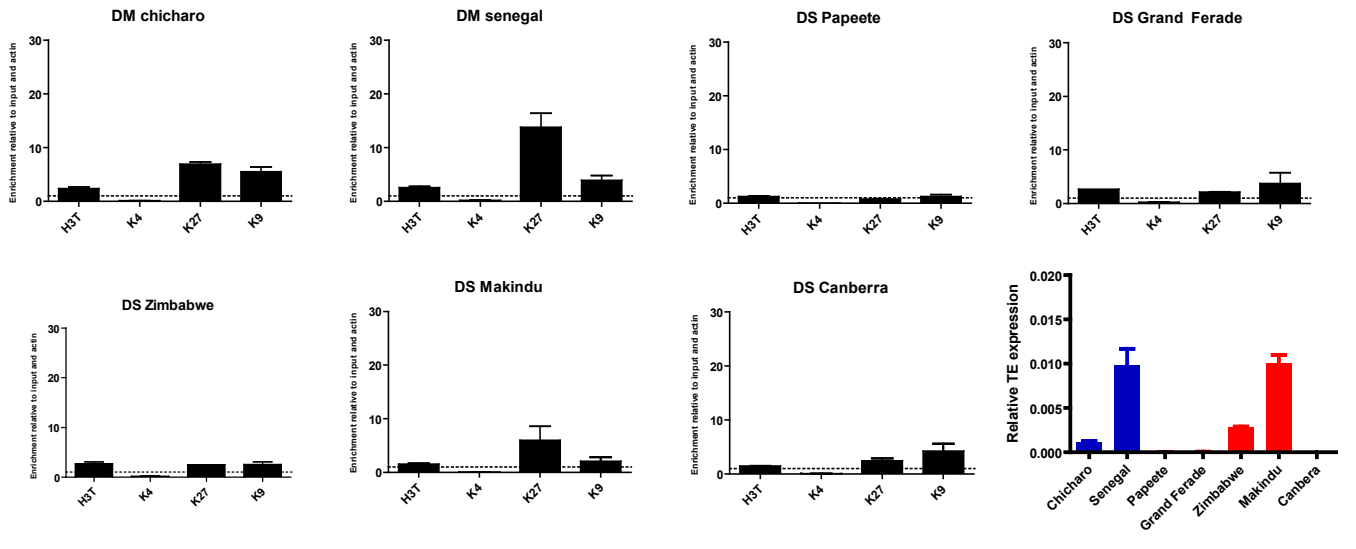

*F*

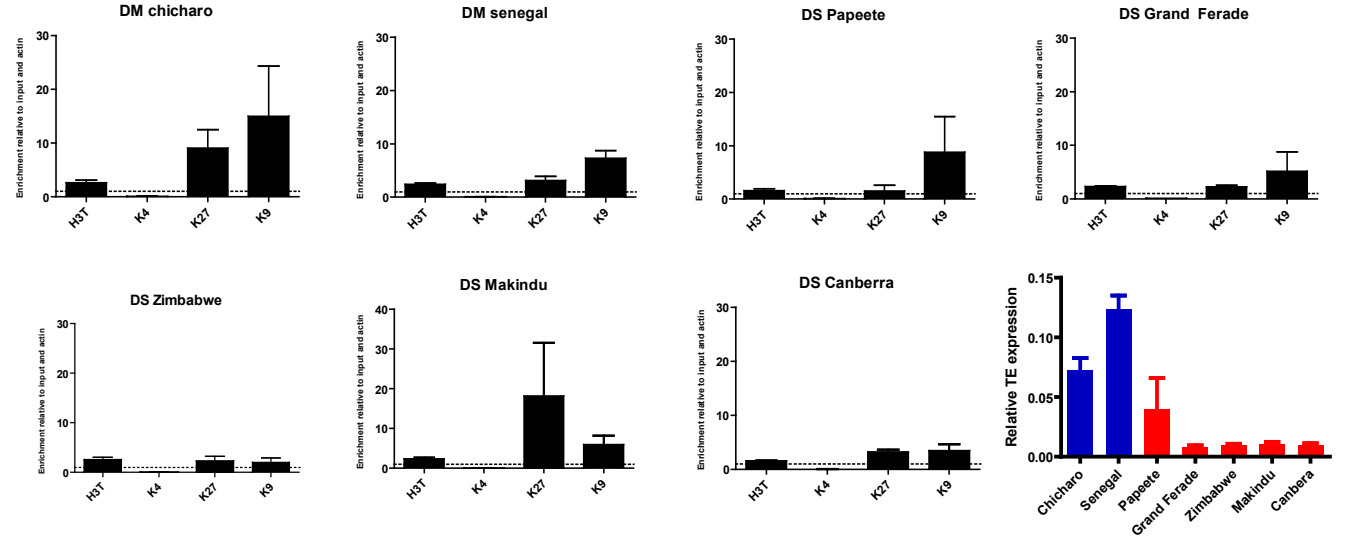

*roo*

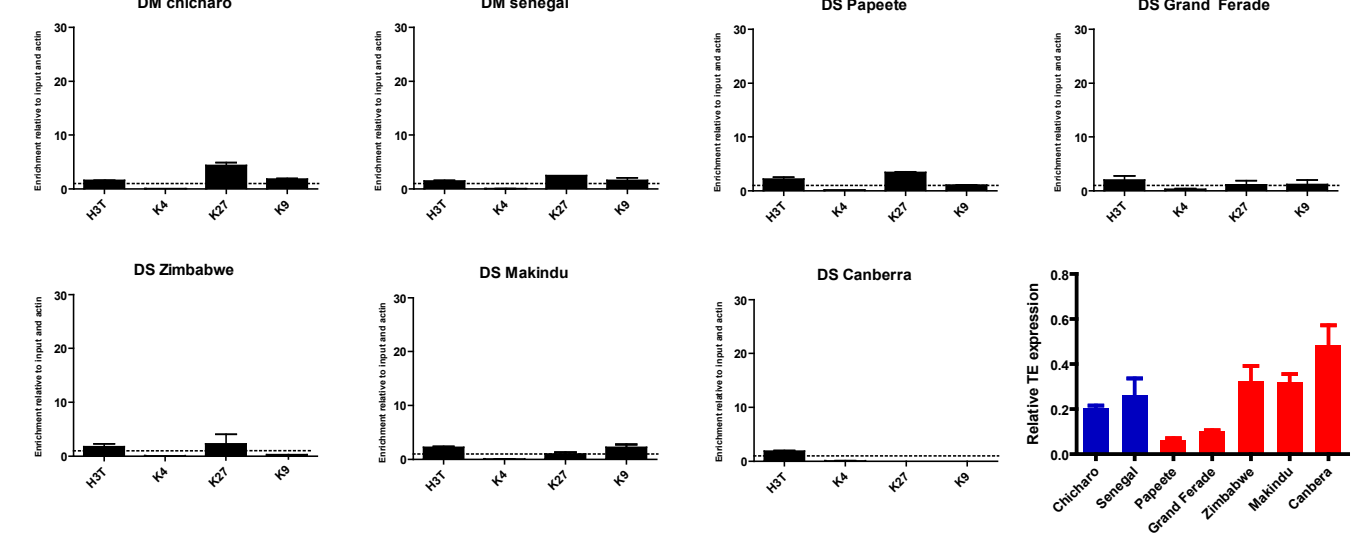

Supplement: Figure S3 — ChIP fold enrichment and transcript analysis for each wild type strain and TE studied (mean ± SE). RT-qPCR data for D. melanogaster (blue) and D. simulans (red). ChIP analysis (black), a dotted line is shown at 1 for no enrichment relative to actin. DM : D. melanogaster, DS : D. simulans. (PDF) [file pone.0044253.s003.pdf]

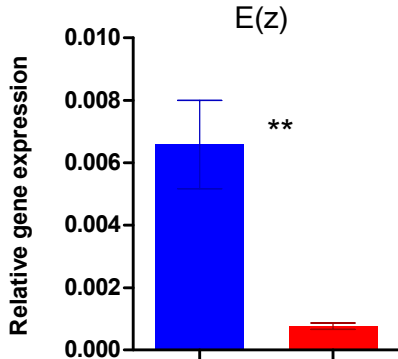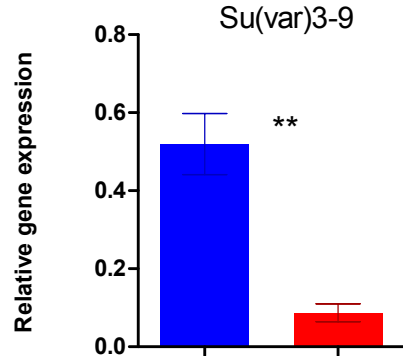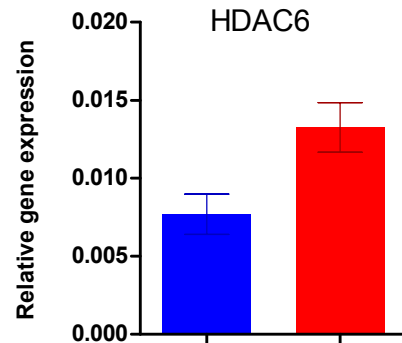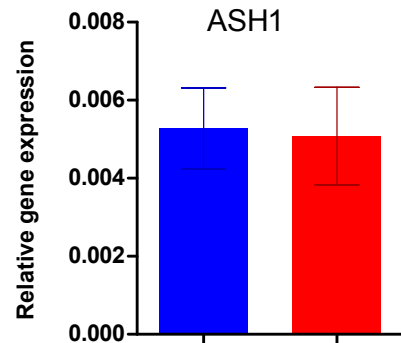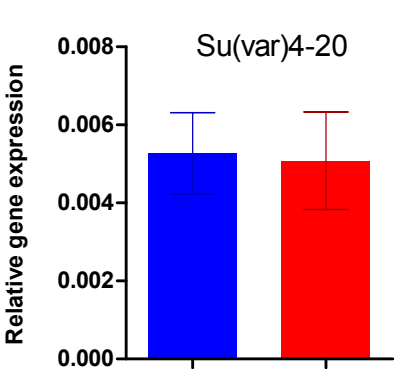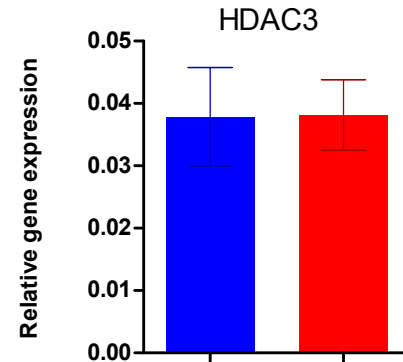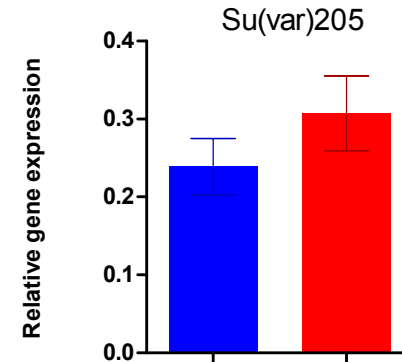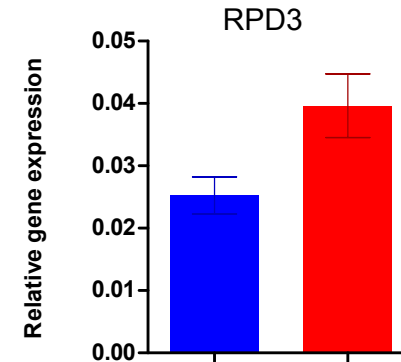

Supplement: Figure S4 — Quantification of RNA steady-state level of epigenetic related enzymes (mean ± SE). D. melanogaster (blue), D. simulans (red). Mann Whitney p-values are shown with asterisks (p-value ** <0.001). (PDF) [file pone.0044253.s004.pdf]

# RNA steady-state level / copy number

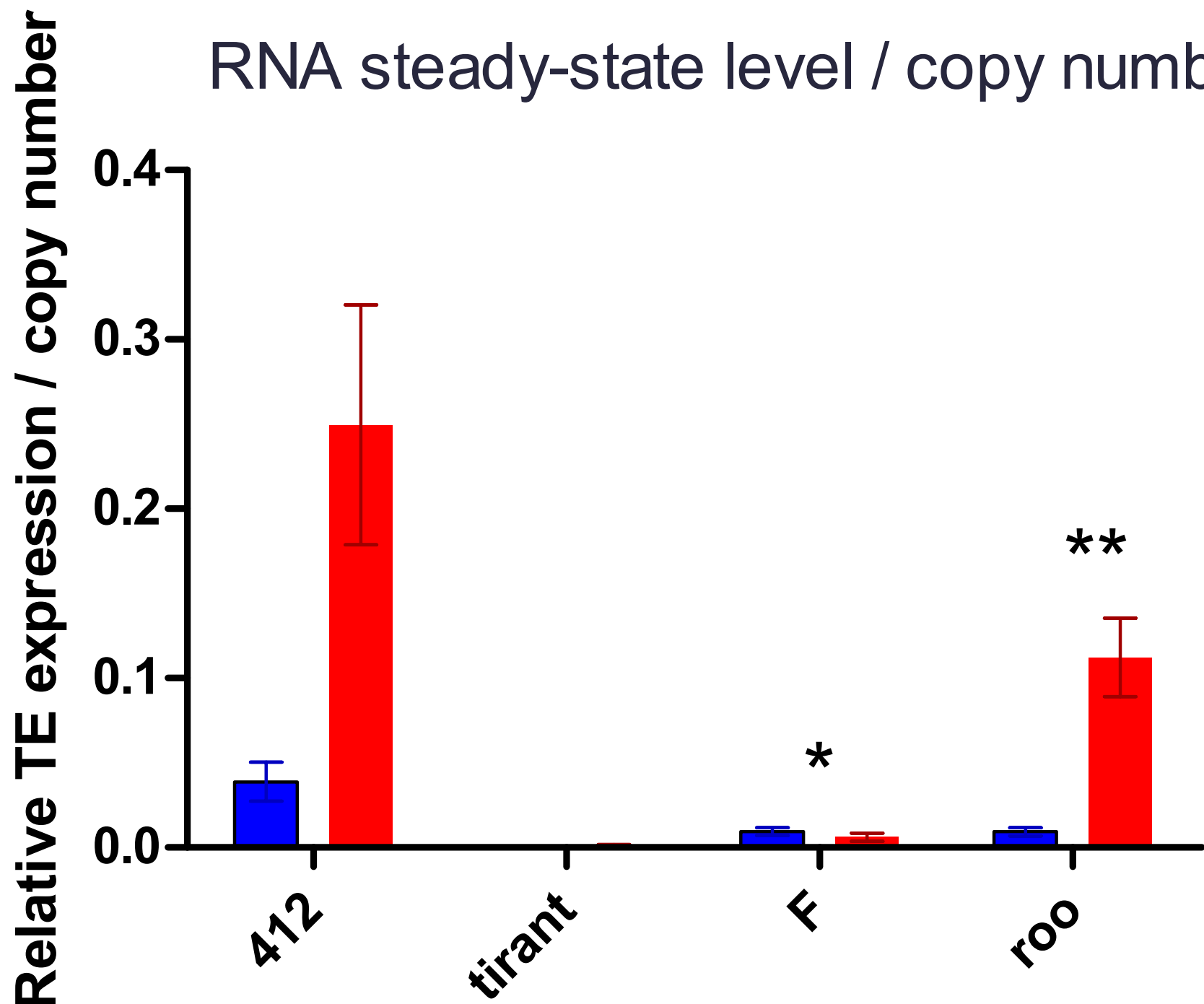

Supplement: Figure S5 — Quantification of TE RNA steady-state level, normalized by copy number (mean ± SE). D. melanogaster (blue), D. simulans (red). Mann Whitney p-values are shown with asterisks (p-value *<0.05, ** <0.001). (PDF) [file pone.0044253.s005.pdf]
